# Laparoscopic management of gastric liposarcoma: A case report and review of the literature

**DOI:** 10.1016/j.ijscr.2020.07.044

**Published:** 2020-07-18

**Authors:** Hector Wadi Cure Jalal, Daniel Gómez, Mauricio Pedraza, Hector Cure Bulicie, Luis Felipe Cabrera, Luis Paolo Gil Gil, David Acevedo, Laura Cabrera, Valery Moreno, Andrés Mendoza

**Affiliations:** a Major in Neuroscience and Minor in Healthcare Management, Chemistry and Psychology, University of Pennsylvania, United States; b Department of General Surgery, Universidad Militar Nueva Granada, Bogotá, Colombia; c Department of General Surgery, Universidad El Bosque, Bogotá, Colombia; d Cirujano endoscopista, Clínica General del Norte, Colombia; e Departament of Surgery José Félix Patiño, Fundación Santa Fe de Bogotá, Universidad de los Andes, Universidad El Bosque, Colombia; f Departament of Surgery, Universidad Metropolitana, Colombia

**Keywords:** Liposarcoma, Diagnostic imaging, Surgery, Endoscopy, Case report

## Abstract

**Introduction:**

Liposarcoma is one of the most common mesenchymal neoplasms in adults. Predominant locations are the retroperitoneum and limbs, it rarely occurs in the gastrointestinal tract. Gastric liposarcoma is extremely rare, with fewer than 30 cases reported around the world.

**Presentation of case:**

Here we present the case of a 70-year-old female patient who was diagnosed with gastric liposarcoma and managed with laparoscopic surgical resection. The patient is currently being followed up and is alive without recurrence 12 months after the operation.

**Discussion:**

Gastric liposarcoma approach requires proper diagnosis with computed tomography and endoscopy, in order to develop an adequate surgical plan. Laparoscopic en-bloc resection and gastrointestinal reconstruction is the ideal management in this type of patient.

**Conclusion:**

Gastric liposarcoma is an extremely rare entity, his management consists of wide en-bloc resection with a gastrointestinal reconstruction.

## Highlights

• Soft tissue tumors are characterized by frequent somatic chromosomal rearrangements.
• Symptoms include epigastric pain, nausea, anorexia, and gastrointestinal bleeding.
• Gastric liposarcoma is the least common sarcoma of the gastrointestinal tract.
• CT and pathological analysis of resected specimens enable diagnosis.
• The gold standard of treatment is surgery with radical resection of the tumor.

## 1 Introduction

Liposarcoma is one of the most common mesenchymal neoplasms in adults, with an incidence of 15–20% of all patients with sarcomas. It generally affects the extremities, the retroperitoneum, and the trunk. Liposarcoma of the gastrointestinal tract is rare, representing only 2% of total cases. To the best of our knowledge, there are less than 50 cases of gastric liposarcoma reported in the world literature [[1], [2], [3]]

Preoperative diagnosis of liposarcoma is often complicated due to the submucosal origin of the tumor. Symptoms are nonspecific or absent in most reported cases. Biopsies obtained through endoscopy are generally negative. Therefore, the definitive diagnosis is obtained through surgical resection and post-surgical histopathological study. The prognosis of this condition is difficult to determine as well. [3,]^4^ We present our experience with the management of gastric liposarcoma, along with a critical analysis of the literature on the diagnostic and therapeutic challenges that this pathology presents. This work has been reported in line with the SCARE criteria [5]

## 2 Presentation of case

A 70-year-old woman was admitted to the emergency department due to a 10-day clinical history comprising abdominal pain in the upper quadrant, without fever, associated with long bowel movements and subjective weight loss. The patient lived in a small town in Colombia with her husband, being a housewife; her drug history of gastritis treated with proton pump inhibitors; she denied any family, allergic, or surgical history. On physical examination, a mass could be palpated in the epigastrium and left hypochondrium, attached to the deep planes. The patient had slight pain on deep palpation, no signs of peritoneal irritation, and absence of adenomegaly. Laboratory tests showed moderate anemia (hemoglobin level 8.5 g/dL); the levels of tumor antigens, electrolytes, and renal function indicators were normal.

**Fig. 1:**
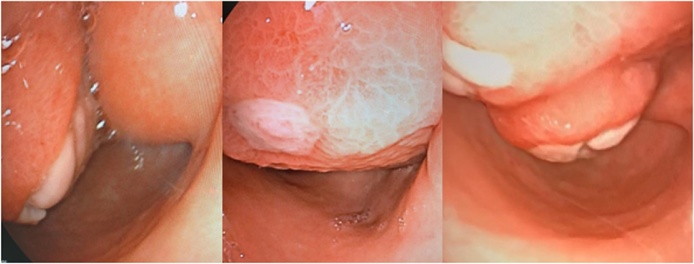
Upper digestive endoscopy: gastric wall with subepithelial lesion with effect of intraluminal mass.

**Fig. 2:**
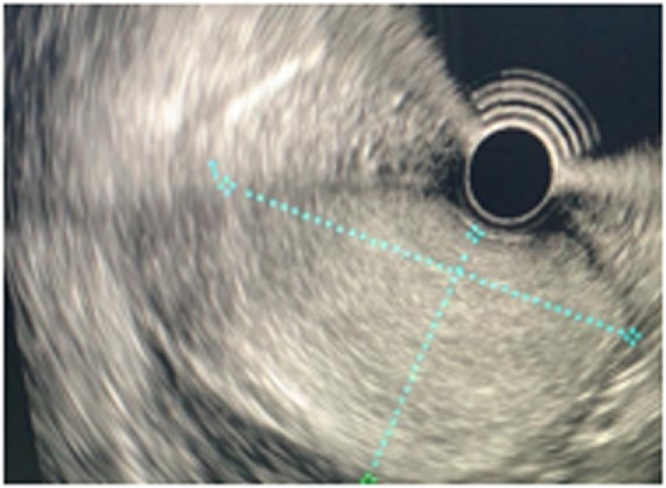
Endoscopic ultrasound: subepithelial lesion with exophytic area.

The abdominal ultrasound showed a mass of 148 × 75 mm in the epigastrium with extension to the left hypochondrium. Endoscopy of the upper digestive tract revealed a subepithelial lesion with a compressive effect on the posterior side of the antrum and duodenum, occupying space in the gastric lumen (Fig. 1). Contrast-enhanced abdominal computed tomography (CT) showed a heterogeneous lesion of the body and antrum with a liquid and fatty component of 57 × 108 mm, with the decreased gastric lumen and a filiform contrast passage with 12-mm peri-gastric lymphadenopathy. Therefore, an endoscopic ultrasound of the stomach and duodenum was performed (Fig. 2), which showed a subepithelial lesion with exophytic and ulcerated area, homogeneous hyperechogenic 12 × 8 cm, dependent on the 3rd echo layer and partial involvement of the muscularis propia, in the gastric greater curvature and antrum. This indicated a gastric tumor of unclear etiology. The patient was therefore scheduled to undergo a radical subtotal gastrectomy by performing laparoscopy with Y-en-Roux reconstruction, accomplished by a laparoscopic surgeon and an interventional surgeon. The surgery proceeded well without perioperative complications and no need for an intensive care unit stay. The patient required only 2 days of hospital stay and tolerated oral intake within the first 24 postoperative hours.

**Fig. 3:**
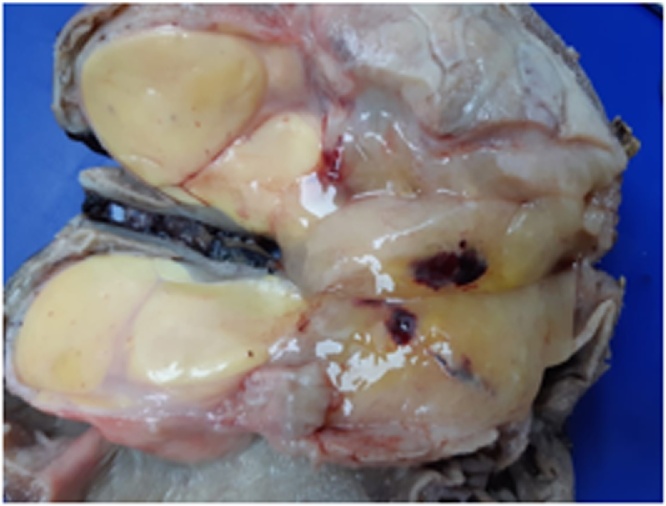
Surgical pathology: gastric liposarcoma.

The pathological analysis reported a lobulated soft mass 15 × 8 × 8 cm covered with centrally ulcerated flattened mucosa. When cut, the mass was solid multi-lobed yellow, had well-defined contours with a nodule larger than 8 × 6 cm, and showed a myxoid appearance (Fig. 3). The microscopic report observed elongated cells in wavy bundles, myxoid areas, and fat differentiation zone accompanying pleomorphic cells with multinucleation, few atypical figures, vacuolated cytoplasm, and acute and chronic inflammation. A total of 19 lymph nodes were removed, all negative for tumor metastases. The final pathological report of a mesenchymal neoplasm with a pleomorphic and myxoid component was compatible with a diagnosis of gastric liposarcoma.

The patient was discharged without any complications. She did not undergo any adjuvant treatment. She remained free of metastasis one year after surgery and is currently being followed up.

## 3 Discussion

Gastric liposarcoma has the highest incidence in patients aged between 50 and 65 years, with the mean patient age being 57 years. It is caused by the proliferation of undifferentiated mesenchymal cells (lipoblasts) within the submucosa and the muscular layer of the stomach. The gastric antrum is the most common location, with 75 % of cases; lesion diameter varies from 1.2–30 cm [1,4] In a study by Frank et al., 1933 cases of liposarcoma were described, of which only 0.31 % were gastric liposarcomas [3], this is the reason why it is crucial to understand the importance of this pathology. This neoplasm can remain asymptomatic for a long time due to its extraluminal growth. The most common clinical symptoms reported in the literature include dyspepsia, nausea, vomiting, anorexia, abnormal bowel movements, asthenia, epigastric abdominal pain, and bleeding from the upper gastrointestinal tract [3]

CT is considered the primary investigation to enable a diagnosis. The presence of areas of fat density is pathognomonic for fatty tumors and the association with areas of improvement is highly suggestive; however, differentiating benign from malignant fatty neoplasms is sometimes difficult due to morphological characteristics. Likewise, CT is the best imaging technique to detect secondary lesions in the liver, lungs, and peritoneum. [3,4] Alternatively, ultrasound can be useful for tumors larger than 2 cm, due to the submucosa of the tumor [1,4] However, the diagnosis is confirmed only with histopathological examination of the surgical specimen. Standard preoperative biopsies are often inadequate due to the submucosal location of the tumor.^4^]

Immunohistochemistry is also useful in the diagnosis of liposarcoma. The expression of MDM2, CDK4 amplification with FISH, and p16 is helpful in differentiating liposarcomas from other adipocytic tumors. Also, fluorescence in situ hybridization in MDM2 amplification is the gold standard to differentiate well-differentiated liposarcomas from lipomas. [6]

Although the published data is limited, surgical resection is the first line of management, due to its good prognosis and the increase in overall survival of patients. According to the sarcoma resection rules, the tumor must be removed with a wide margin of 5 cm of healthy tissue around it. There is currently no evidence that chemotherapy or radiotherapy improves overall survival rates in this group of patients [4]

In this case, an endoscopic submucosal resection was not considered due to the size of the tumor and the perforation risk [6].

Various classification systems have been developed to differentiate between low- and high-grade tumors. In practice, two classification systems are used: the NCI (National Cancer Institute) classification and the FNCLCC (Fédération Nationale des Centers de Lutte Contre le Cancer). [3] According to the 2013 classification of the World Health Organization (WHO), liposarcoma is divided into 5 types: Atypical lipomatous / well-differentiated tumor, dedifferentiated, myxoid, pleomorphic, and liposarcoma without other specification. The most common type is the well-differentiated (40 %–45 %); it is usually low-grade, slow-growing, and with a low probability of metastasis. The undifferentiated type is a high-grade sarcoma. It is very common in the retroperitoneal región [7]; they usually have areas of hemorrhage and necrosis together with solid areas. Due to this, it can be misdiagnosed as the pleomorphic type. The pleomorphic type of liposarcoma usually originates from a well-differentiated. The myxoid type (35 %) shows a myxoid matrix with abnormal-looking cells, shows an intermediate level of aggressiveness. This type has a variation called "round cell" that is more at risk of metastasis. Pleomorphic liposarcoma is the least common type; it is highly malignant and shows high mitotic activity, hemorrhage, and necrosis [1]. Mortality can reach 80 % for visceral or retroperitoneal tumors [4].

Differential diagnosis of gastric liposarcomas includes lipoma, peritoneal liposarcoma, carcinoma of the perivisceral fat, gastric stromal tumors, liver metastases adherent to the stomach, peritoneal carcinomatosis, and primary tumor of the omentum. [8,9]

## 4 Conclusion

Gastric liposarcoma is a rare pathology, without a clear incidence and with atypical symptoms. However, it is important to have diagnostic suspicion based on tomographic findings and offer radical surgery. Therefore, it is important to continue reporting each case in the world literature and to collect further data regarding this condition.

## Conflicts of interest

The authors declare not having any conflict of interests, any financial or personal relationships with other people or organisations that could inappropriately influence our work

## Funding

The authors declare no funding associated with the collection and analysis of data as with writing or submission of the article.

## Ethical approval

The study is exempt from ethnical approval in our institution.

## Consent

Written informed consent was obtained from the patient for publication of this case report and accompanying images

## Author contribution

Hector Wadi Cure Jalal: Data collection

Daniel Gómez: Data collection

Mauricio Pedraza: Data collection, data analysis and interpretation writing the paper

Hector Cure Bulicie: Study design, data analysis or interpretation

Luis Felipe Cabrera: Data collection, data analysis and interpretation, writing the paper

Luis Paolo Gil Gil: Study design, data analysis or interpretation

David Acevedo: writing the paper

Laura Cabrera: writing the paper

Valery Moreno: writing the paper

Andrés Mendoza: writing the paper

## Registration of research studies

N/A.

## Guarantor

Mauricio Pedraza Ciro, Luis Felipe Cabrera Vargas

## Provenance and peer review

Not commissioned, externally peer-reviewed
